# Predicting the risk of active pulmonary tuberculosis in people living with HIV: development and validation of a nomogram

**DOI:** 10.1186/s12879-022-07368-5

**Published:** 2022-04-19

**Authors:** Jinou Chen, Ling Li, Tao Chen, Xing Yang, Haohao Ru, Xia Li, Xinping Yang, Qi Xie, Lin Xu

**Affiliations:** 1 Division of Tuberculosis Control and Prevention, Yunnan Center for Disease Control and Prevention, Kunming, China; 2 Family Health International Office, Kunming, China; 3 Yunnan Provincial Hospital of Infectious Disease, Kunming, China

**Keywords:** Tuberculosis, HIV, Nomogram

## Abstract

**Background:**

Diagnosis of pulmonary tuberculosis (PTB) among people living with HIV (PLHIV) was challenging. The study aimed to develop and validated a simple, convenient screening model for prioritizing TB among PLHIV.

**Methods:**

The study included eligible adult PLHIV participants who attended health care in Yunnan, China, from January 2016 to July 2019. Participants included before June 2018 were in the primary set; others were in the independent validation set. The research applied the least absolute shrinkage and selection operator regression to identify predictors associated with bacteriological confirmed PTB. The TB nomogram was developed by multivariate logistic regression. The C-index, receiver operating characteristic curve (ROC), the Hosmer–Lemeshow goodness of fit test (H–L), and the calibration curves were applied to evaluate and calibrate the nomogram. The developed nomogram was validated in the validation set. The clinical usefulness was assessed by cutoff analysis and decision curve analysis in the primary set.

**Result:**

The study enrolled 766 PLHIV, of which 507 were in the primary set and 259 in the validation set, 21.5% and 14.3% individuals were confirmed PTB in two sets, respectively. The final nomogram included 5 predictors: current CD 4 cell count, the number of WHO screen tool, previous TB history, pulmonary cavity, and smoking status (*p* < 0.05). The C-statistic was 0.72 (95% CI 0.66–0.77) in primary set and 0.68 (95% CI 0.58–0.75) in validation set, ROC performed better than other models. The nomogram calibration was good (H–L *χ*^2^ = 8.14, *p* = 0.15). The area under the decision curve (0.025) outperformed the existing models. The optimal cutoff for screening TB among PLHIV was the score of 100 (sensitivity = 0.93, specificity = 0.35).

**Conclusion:**

The study developed and validated a discriminative TB nomogram among PLHIV in the moderate prevalence of TB and HIV. The easy-to-use and straightforward nomogram would be beneficial for clinical practice and rapid risk screening in resource-limited settings.

**Supplementary Information:**

The online version contains supplementary material available at 10.1186/s12879-022-07368-5.

## Background

The prevalence of human immunodeficiency virus (HIV) and coinfection with tuberculosis (TB) was challenging to control the HIV and TB epidemic. TB was a critical opportunistic infection for people living with HIV (PLHIV). HIV-related TB had an incremental risk and derived disease burden globally [1]. The World Health Organization (WHO) estimated 815,000 TB incidence among HIV-positive people with 208,000 death in 2019, while the number was 14,000 and 2200 in China [2].

Regular screening for active pulmonary tuberculosis (PTB) in PLHIV, rapid PTB diagnosis are the key points for controlling the TB/HIV coinfection, also crucial to stop PTB transmission and reduce mortality among the susceptible population [3–5]. However, the diagnosis of PTB among PLHIV was particularly challenging. TB/HIV co-morbidity individuals might have atypical, non-specific clinical features and often a smear-negative disease [6]. WHO developed a symptom-based screening tool for intensifying PTB case detection in PLHIV, and it was recommended as routine work under the national TB program (NTP) to prioritize TB for isoniazid preventive therapy (IPT) in the low-resource setting [7, 8]. The WHO-recommended four symptom screen (W4SS) could identify PLHIV as having a high probability of PTB disease with one of four symptoms: current cough, fever, weight loss, or night sweat. The latest evidence of WHO guidelines showed that W4SS has relatively high sensitivity in adults and adolescents living with HIV, 83%, but low specificity, 38%. The sensitivity of W4SS among outpatients on ART was relatively low, 53%, indicating that W4SS alone would not be sufficient to detect TB among people in regular ART care. W4SS was relatively sensitive in outpatients not on ART (84%), indicating that W4SS is useful in finding people with TB among those starting HIV care, but the lack of specificity has implications for resources and rational use of diagnostic testing [9, 10].

The molecular test was applied for diagnosis of TB among PLHIV to improve the yield and speed of diagnosis, included the GeneXpert MTB/RIF assay (Cepheid, Sunnyvale, USA) (Xpert), and updated the Xpert Ultra (Ultra) [11–14]. WHO endorsed and recommended using the molecular WHO-recommended rapid diagnostics (mWRDs) for TB screening among inpatient HIV in medical wards where TB prevalence over 10% [15]. The challenge was Xpert equipment and reagent remains unavailable in primary health care units under resource-limited setting. Meanwhile, the symptom-based screening rule generated a large number of PLHIV without active PTB. The sequential screening strategy of W4SS algorithm then Xpert test was impractical. Thus, there was the need to predict the highest risk, prioritize the PTB screen, and sustain the resource.

Previous studies developed the multivariable prediction model (MPM) to calculate the probability of TB disease or improve TB case finding among PLHIV [16–18]. Although the researches were conducted in high TB and HIV prevalence locations, models could not validate in different TB/HIV prevalence settings. Furthermore, many predictors of the models were unavailable for resource-limited settings.

This study aimed to develop, validate, and assess a practical screening nomogram that integrated demographics, symptoms, and clinical factors. In addition, to determine whether the nomogram provides more accurate prediction and utility of the detection for bacteriological confirmed PTB among PLHIV in a moderate TB and HIV prevalence setting.

## Methods

### Study setting and participants

Yunnan province located southwest of China, shares a border with Myanmar, Laos, and Vietnam, with a moderate TB and HIV prevalence [19]. We developed and validated our nomogram applied the operational research data, a cross-sectional study that evaluates bacteriological confirmed PTB among PLHIV in Yunnan Provincial Hospital of Infectious Disease.

The study consecutively recruited eligible participants as a primary set between January 2016 and June 2018 in the study site. Then an independent set included eligible participants as a validation set between July 2018 and July 2019. The eligible participants in the primary and validate set were included or excluded using the same criteria, and they followed the identical investigation and laboratory testing.

The participants who met the inclusion criteria were recruited as eligible patients: inpatient or outpatient infected with HIV or diagnosed AIDS patients regardless of ART status and previously TB; age over 15 years; any symptomatic PLHIV, suitable for the TB screening and testing without critical or severe illness; and gave written informed consent to participate in the study. The exclusion criteria were as follows: refused to participate; did not give writing consent in the survey; current under anti-TB treatment; pregnant and maternal woman; abnormal mental condition, severe or emergency condition.

This study was conducted and reported by following the Transparent Reporting of a multivariable prediction model for Individual Prognosis Or Diagnosis (TRIPOD) guideline developing, validating, or updating a prediction model [20]. TRIPOD checklist was displayed in Additional file 1: Table S1.

### Study outcome

We defined the study outcome as bacteriological confirmed PTB and not PTB among PLHIV. Four sputum samples were requested when submitting a sputum test (two instant spot sputums, the third at night, and the fourth in the following morning). The bacteriological confirmed PTB was those PLHIV have any of the positivity of laboratory result: (1) Xpert MTB/RIF or (2) smear-positive or (3) *M. tuberculosis* culture-positive from any of the sputum samples. The laboratory procedure was stated in Additional file 2: Method S1.

### Predictors

Potential predictors associated with PTB among PLHIV were pre-specified based on epidemiological, clinical experience, and current literature review [21–26]. The factors included age, sex, body mass index (BMI, kg/m^2^), ART status (Pre-ART, On-ART), current CD 4 cell count (cells/ul), current cough (yes, no), fever (yes, no), unintentional weight loss (yes, no), night sweats (yes, no), hemoptysis (yes, no), tired (yes, no), the number of WHO screen tool of TB symptoms (current cough, fever, weight loss, and night sweats), previous TB history (yes, no), abnormal chest radiograph (yes, no), pulmonary cavity (yes, no), smoking status (never smoked, current smoking), alcohol use (never drank, current drinking). The following factors were self-reported by participants: age, TB symptoms, previous TB history, smoking status, and alcohol use; the rest were from the medical examination, test, or investigation.

### Sample size calculation

The calculation of study sample size followed the method of developing a clinical prediction model [27, 28]. The parameters of C-index were set as 0.8, the TB/HIV prevalence was estimated as 0.2 [29], number of predictors was 17, the calculation resulted the minimum sample size was 752.

### Statistical analysis

All statistical analysis was conducted with R software 3.6.2 (http://www.Rproject.org). The level of *p* < 0.05, two-sided was set as statistical significance.

Demographic and clinical characteristics were grouped based on the study outcome. The categorical variables were summarized as frequency and compared using the chi-square test and Fisher’s exact test as appropriate. The continuous variables were presented as the median and interquartile range (IQR).

### Predictor selection

The least absolute shrinkage and selection operator (LASSO) method [30, 31], which suitable for the multiple variable selection and regression in high-dimension or highly correlated data, was applied to select the predictive features in the primary set. The 1-SE criteria defined the best LASSO parameter *λ*, which the regularized model that the ten-fold cross-validated error was within one standard error of the minimum. The optimal LASSO shrinkage parameter *λ* was in the 1-SE criteria with least number variables. The LASSO coefficient trace plot against variables determined the nonzero coefficients and variables analyzed in next model build step.

### Development of the nomogram

The association between LASSO selected variables and the study outcome was assessed by univariate binary logistic regression. The multivariate prediction model was performed by using LASSO-selected variables. Based on the multivariate logistic regression results in the primary set, we built a quantitative and scoring tool of a nomogram to predict the PLHIV individual probability of PTB diagnosis. The scores of variables in nomogram were defined by ratio method. The score of variable *i* could be calculated as ${score}_{i}={\beta }_{i}\frac{100}{{\beta }_{max}}$, where the ${\beta }_{i}$ was regression coefficient of variable *i*, ${\beta }_{max}$ was the max $\beta$ of all regression coefficients, the score of max $\beta$ variable was set as 100. Total score of nomogram was defined as ${score}_{total}=\sum_{i=1}^{n}{score}_{i}$.

### Model evaluation and calibration

Harrell’s C-index assessed the performance of the nomogram. The C-index represented the discriminative ability; the larger C-index, the more accurate the prediction was. Meanwhile, the sensitivity and specificity of nomogram were performed with receiver operating characteristic curve (ROC). The previous XPHACTOR model (predictors included: BMI, ART status, current CD 4 cell count, the number of WHO screen tool of TB symptoms) [17], and W4SS algorithm was compared the area under the curve (AUC) with the tuberculosis nomogram.

The Hosmer–Lemeshow goodness of fit test (H–L test) was applied for model calibration; a nonsignificant statistic of the H–L test implied the nomogram calibrated perfectly [32–34]. To assess the agreement between actual and predicted outcomes, we generated calibration curves to evaluate the nomogram. The in-sample and a bootstrapping validation (1000 bootstrap samples) were applied for depicted calibration curves.

### Model validation

The independent validation was conducted in the validation set to test the generalization performance of the nomogram. The data of validation set were reintroduced into the previous established nomogram for internal validation. The performance of the nomogram in the validation set was assessed by C-index, ROC analysis, AUC comparison, H–L test, and calibration curves, as stated before.

### Clinical utility

The cutoff analysis was performed to evaluate the applicability of the nomogram. The continuous scores and corresponding cutoffs were depicted with sensitivity and specificity of nomogram, aimed to find the optimal threshold for screening TB among PHILV. Decision curve analysis (DCA) was performed to evaluate the clinical usefulness of the nomogram. The clinical usefulness was defined as a net benefit (summing the true positives diagnosis and subtracting the weighted risk of false positives diagnosis) at sequential threshold probability. Thus, the balanced net benefit was the benefit of PLHIV with TB started empiric treatment (true positives) minus the harm of PHLHV without TB began empiric treatment (false positives) under various thresholds. We compared the net benefit of our nomogram to the previous XPHACTOR model and W4SS algorithm. A net benefit value of 0 meant no benefit of the model; higher values indicate more benefit [35, 36]. The benefit–cost ratio and area under the decision curve (AUDC) were used to evaluate the clinical utility of the risk model.

### Sensitivity analysis

To test the stability and applicability of the model in different settings, we thus conducted sensitivity analysis based on the developed nomogram. By introducing and substituting variables in the model, we assessed the sensitive model with C-index, H–L test, and DCA analysis.

## Results

### Study population and characteristics

In total, the study enrolled 766 PLHIV for investigation and examination, of which 66.2% (507/766) were in the primary set and 33.8% (259/766) in the validation set, respectively. The primary set identified 21.5% (109/507) bacteriological confirmed individuals and 78.5% (398/507) participants with negative test results; the proportion was 14.3% (37/259) and 85.7% (222/259) for the validation set.

PLHIV characteristics and the comparisons were shown in Table 1. The primary set consisted of 373 males and 134 females; the median age was 45 years. The validation set comprised 178 males and 81 females; the median age was 45 years. Ten of the factors in the primary set was significantly associated with bacteriological confirmed PTB, included the ART status, current CD 4 cell count, current cough, night sweats, unintentional weight loss, the number of WHO screen tool, previous TB history, abnormal chest radiograph, pulmonary cavity, and smoking status (*p* < 0.05).

**Table 1:** Baseline and clinical characteristics of primary and validation population

Characteristics	Primary set *N* (%)			Validation set *N* (%)		
	Confirmed PTB cases	Not PTB	*p*	Confirmed PTB cases	Not PTB	*p*
All	109 (21.5%)	398 (78.5%)		37(14.3%)	222(85.7%)	
Sex						
Female	88 (80.7%)	285 (71.6%)	0.06	23 (62.2%)	155 (69.8%)	0.35
Male	21 (19.3%)	113 (28.4%)		14 (37.8%)	67 (30.2%)	
Age (years)						
Median (IQR)	43(37–49)	45(37–55)		42(31–52)	45(36–56)	
< 25	3 (2.8%)	9 (2.3%)	0.29	2 (5.4%)	3 (1.4%)	0.28
25–44	59 (54.1%)	176 (44.2%)		19 (51.4%)	101 (45.5%)	
45–64	38 (34.9%)	170 (42.7%)		12 (32.4%)	89 (40.1%)	
≥ 65	9 (8.3%)	43 (10.8%)		4 (10.8%)	29 (13.1%)	
BMI (kg/m^2^)						
Median (IQR)	18.9(17.2–20.6)	19.9(17.7–22.0)		17.9(16.9–19.8)	20.0(18.0–21.9)	
< 18.5	44 (40.4%)	146 (36.7%)	0.38	19 (51.4%)	72 (32.4%)	0.08
18.5–24	59 (54.1%)	214 (53.8%)		15 (40.5%)	127 (57.2%)	
≥ 24	6 (5.5%)	38 (9.5%)		3 (8.1%)	23 (10.4%)	
ART status						
On ART	44 (40.4%)	221 (55.5%)	< 0.01*	15 (40.5%)	143 (64.4%)	< 0.01*
Pre-ART	65 (59.6%)	177 (44.5%)		22 (59.5%)	79 (35.6%)	
Current CD 4 cell count (cells/μl)						
Median (IQR)	52 (32–114)	119 (44–298)		84 (31–158)	151 (50–343)	
< 100	73 (67.0%)	176 (44.2%)	< 0.01*	22 (59.5%)	90 (40.5%)	< 0.01*
100–199	16 (14.7%)	74 (18.6%)		10 (27.0%)	38 (17.1%)	
≥ 200	20 (18.3%)	148 (37.2%)		5 (13.5%)	94 (42.3%)	
Symptoms						
Cough (yes)	79 (72.5%)	226 (56.8%)	< 0.01*	22 (59.5%)	92 (41.4%)	0.04*
Fever (yes)	66 (60.6%)	204 (51.3%)	0.08	21 (56.8%)	77 (34.7%)	0.01*
Weight loss (yes)	62 (56.9%)	164 (41.2%)	< 0.01*	22 (59.5%)	96 (43.2%)	0.06
Night sweat (yes)	54 (49.5%)	128 (32.2%)	< 0.01*	16 (43.2%)	60 (27.0%)	0.04*
Hemoptysis (yes)	6 (5.5%)	13 (3.3%)	0.26	3 (8.1%)	5 (2.3%)	0.09
Tired (yes)	34 (31.2%)	154 (38.7%)	0.15	11 (29.7%)	86 (38.7%)	0.29
No. of WHO symptoms						
0	13 (11.9%)	90 (22.6%)	< 0.01*	7 (18.9%)	80 (36.0%)	0.04*
1	17 (15.6%)	75 (18.8%)		5 (13.5%)	44 (19.8%)	
2	28 (25.7%)	106 (26.6%)		7 (18.9%)	42 (18.9%)	
3	16 (14.7%)	73 (18.3%)		10 (27.0%)	27 (12.2%)	
4	35 (32.1%)	54 (13.6%)		8 (21.6%)	29 (13.1%)	
Previous TB history						
Yes	28 (25.7%)	55 (13.8%)	< 0.01*	8 (21.6%)	40 (18.0%)	0.64
No	81 (74.3%)	343 (86.2%)		29 (78.4%)	182 (82.0%)	
Abnormal chest radiograph						
Yes	95 (87.2%)	306 (76.9%)	0.02*	34 (91.9%)	166 (74.8%)	0.02*
No	14 (12.8%)	92 (23.1%)		3 (8.1%)	56 (25.2%)	
Pulmonary cavity						
Yes	12 (11.0%)	15 (3.8%)	< 0.01*	5 (13.5%)	3 (1.4%)	< 0.01*
No	97 (89.0%)	383 (96.2%)		32 (86.5%)	219 (98.6%)	
Smoking status						
Never	50 (45.9%)	263 (66.1%)	< 0.01*	21 (56.8%)	128 (57.7%)	0.91
Current	59 (54.1%)	135 (33.9%)		16 (43.2%)	94 (42.3%)	
Alcohol use						
Never	86 (78.9%)	330 (82.9%)	0.33	28 (75.7%)	171 (77.0%)	0.84
Current	23 (21.1%)	68 (17.1%)		9 (24.3%)	51 (23.0%)	

*p value < 0.05

*PTB* pulmonary tuberculosis, *IQR* interquartile range, *BMI* body mass index, *ART* antiretroviral therapy, *WHO* World Health Organization

### Predictor selection

**Fig. 1:**
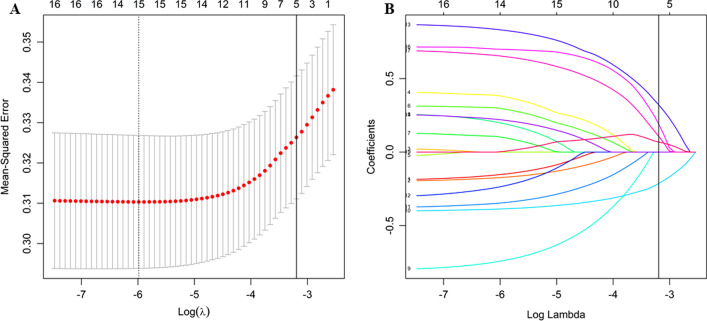
Feature selection applying the least absolute shrinkage and selection operator with binary logistic regression model. **A** LASSO shrinkage parameter *λ* selected by 10- fold cross-validation with minimum criteria. The Mean-Squared Error (red dots) and its 95% error bar (grey interval) was plotted against ln(*λ*) sequence. Vertical line shown the optimal value of *λ* selected by the minimum criteria (1-SE criteria: the regularized model that the cross-validated error is within one standard error of the minimum). A shrinkage factor *λ* of 0.041, with ln(*λ*) = − 3.19 was chosen for optimal parameter. **B** LASSO coefficient trace of features. LASSO coefficient trace was plotted against the ln(*λ*) sequence, vertical line was drawn by the optimal *λ* value selected in the first step, where result in 5 nonzero coefficients (current CD 4 cell count, the number of WHO screen tool of TB symptoms, pulmonary cavity, previous TB history and smoking status) with the minimum criteria. *LASSO* least absolute shrinkage and selection operator, *MSE* mean-squared error, *TB* tuberculosis

The LASSO logistic regression model returned five nonzero coefficients while choosing the minimum criteria optimal shrinkage factor (Fig. 1). Based on the primary set, the collected 17 features were reduced to 5 predictors for the next step model development: current CD 4 cell count, the number of WHO screen tool, previous TB history, pulmonary cavity, and smoking status.

### Development of prediction model

**Fig. 2:**
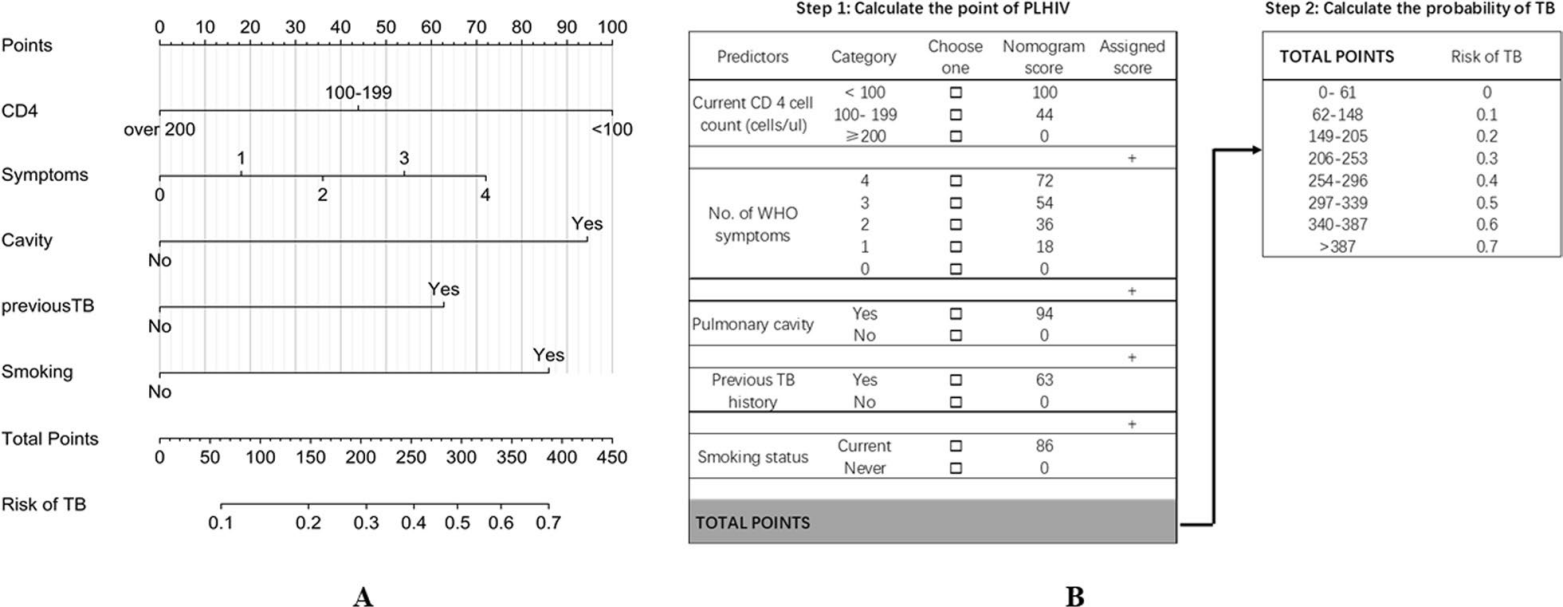
Tuberculosis nomogram and risk calculator among people with HIV/AIDS. **A** Tuberculosis nomogram for people with HIV/AIDS. **B** Tuberculosis risk calculator for people with HIV/AIDS. To use the nomogram in **A**, an individual PLHIV category located on each predictor axis, which corresponding to the Points axis overhead, a vertical line could be drawn to determine the score of each variable. The summation score of 5 variables located on the Total Points axis, a vertical line could be drawn downward to the Risk of TB axis to determine the likelihood of the bacteriologically confirmed tuberculosis in PLHIV. The risk calculation was followed the steps in **B**. First, define and choose the nomogram scores corresponding to the PLHIV individual category, then sum up all five assigned scores, and got the total points of PLHIV individual. Second, find the risk of bacteriologically confirmed tuberculosis in PLHIV in the right-hand side table corresponding the total points. *PLHIV* people with HIV/AID; *TB*, tuberculosis

Table 2 summarized the development of the risk prediction model. The table showed that the predictors included current CD 4 cell count, the number of WHO screen tool, pulmonary cavity, previous TB history, and smoking status had a significant relationship with confirmed PTB (*p* < 0.05). All predictors were fitted into the multivariable binary logistic regression and shown as independent predictors. The TB nomogram that integrated all the above significant predictors for the primary set was developed and presented in Fig. 2A. The practical step-by-step TB risk calculator among PLHIV was showed in Fig. 2B. Additional file 3: Table S2 showed the calculation to define the points of variables.

**Table 2:** Binary logistic regression model for predicting bacteriological confirmed tuberculosis in people living with HIV

Factors	Unadjusted Odds Ratio	95% CI	*p*	Adjusted Odds Ratio	95% CI	*p*
CD 4 count (< 100 *vs.* ≥ 200)	3.07	(1.82–5.39)	< 0.01	2.55	(1.44–4.64)	< 0.01
CD 4 count (100- 199 *vs.* ≥ 200)	1.60	(0.77–3.26)	0.19	1.51	(0.72–3.14)	0.27
No. of WHO symptoms	1.38	(1.17–1.62)	< 0.01	1.18	(1.01–1.42)	0.05
Pulmonary cavity (yes *vs.* no)	3.16	(1.41–6.96)	< 0.01	2.42	(1.03–5.57)	0.04
Previous TB history (yes *vs.*no)	2.16	(1.28–3.59)	< 0.01	1.80	(1.03–3.10)	0.04
Smoking status (current *vs.* never)	2.30	(1.50–3.54)	< 0.01	2.23	(1.43–3.51)	< 0.01

*WHO* World Health Organization, *TB* tuberculosis

**Fig. 3:**
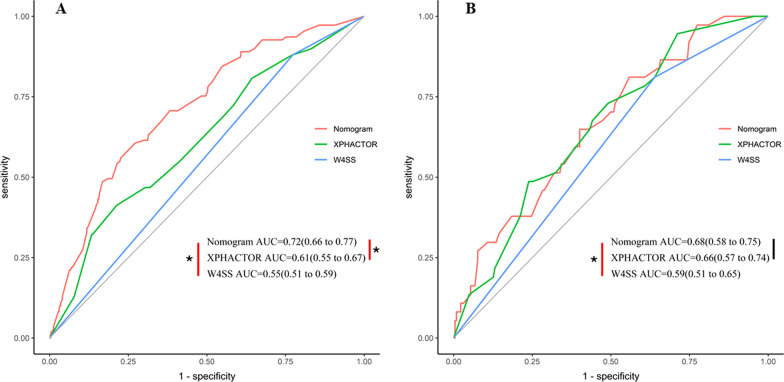
Receiver operating characteristic area under the curve showing the discriminative performance of 3 different algorithms. **A** Predictive performance for the primary set. **B** Predictive performance for the validation set. The red line represents the ROC and AUC of tuberculosis nomogram, The green line represents the ROC and AUC of XPHACTOR study rule (included ART status, CD4 count, BMI, and the number of WHO symptoms), The blue line represents the ROC and AUC of W4SS algorithm. The vertical lines show the comparison between different algorithm AUCs, the red vertical lines with asterisk were AUC difference significant *p* < 0.05, the black vertical line was insignificant and *p* > 0.05. *ROC* receiver operating characteristic curve, *AUC* area under the curve, *W4SS* WHO-recommended four symptom screen

**Fig. 4:**
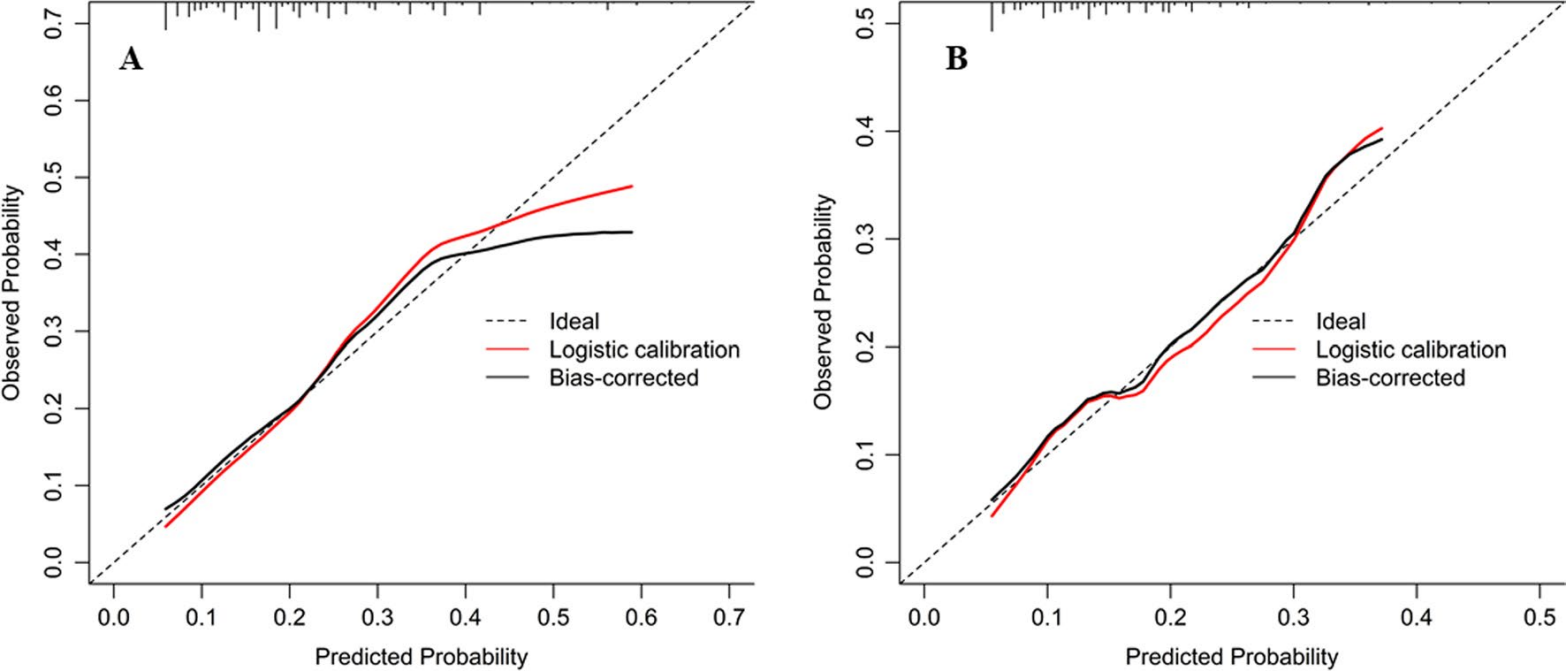
Calibration curve of the tuberculosis nomogram. **A** Calibration curve of the tuberculosis nomogram in the primary set. **B** Calibration curve of the tuberculosis nomogram in the validation set. The calibration curve represents the agreement between nomogram-predicted risk of confirmed TB and actual diagnosis outcome. The spike histogram at the top shows the distribution of predicted risks. The dash line in diagonal shows a perfectly-calibrated model. Red and black solid lines show the nomogram in-sample calibration outcome and the bias-corrected calibration by bootstrap resampling 1000 times respectively

The C-index of the PTB model among PLHIV in the primary set was 0.72 (95% CI, 0.66–0.77). The AUC of ROC for the nomogram (0.72) was bigger than XPHACTOR model (0.61) and W4SS algorithm (0.55, *p* < 0.05, Fig. 3A). The H–L test showed a nonsignificant statistic (*χ*^*2*^ = 8.14, *p* = 0.15), suggested that the nomogram performance was no departure from the ideal fit. The calibration plot showed that it along with the diagonal, though over prediction presented in the high-risk section (Fig. 4A).

### Validation of the nomogram

The independent validation of nomogram performed in the validation set, the C-index of the PTB nomogram was 0.68 (95% CI, 0.58–0.75). The AUC of ROC for nomogram (0.68) was bigger than W4SS algorithm (0.59, *p* < 0.05), but it was identical with XPHACTOR model (*p* > 0.05, Fig. 3A). The calibration curve presented a favorable overall agreement between the observed and predicted probability of PTB (Fig. 4B).

### Clinical utility

**Fig. 5:**
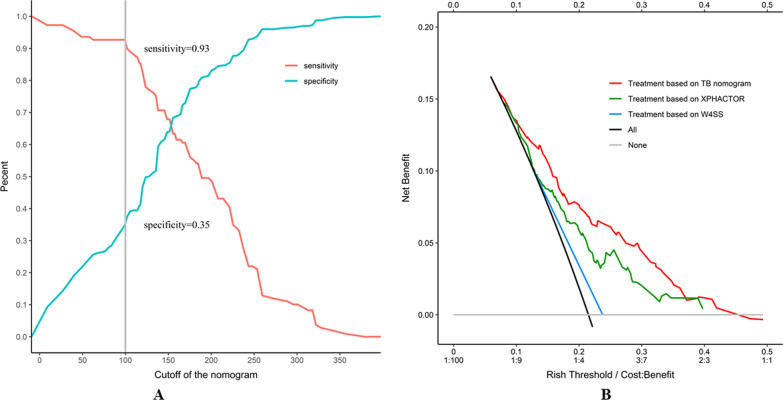
Performance of cutoffs and decision curve analysis for tuberculosis diagnostic nomogram among people with HIV/AIDS. **A** The sensitive and specific performance of the tuberculosis nomogram at different cut-offs. **B** Decision curve analysis compares the standardized net benefit of different strategies. The clinical utility of four diagnostic strategies is compared by plotting the net benefit (y axis) for the threshold based on the risk of bacteriological confirmed tuberculosis (x axis). Net benefit calculated by summing the benefits (true positives) and subtracting the harms (false positives, weighting by the relative harm of withdraw treatment and an unnecessary treatment). The red line represents the developed nomogram yielded highest net benefit and area under decision curve for potential thresholds (ranging from 10 to 41%). The green line represents the net benefit of XPHACTOR study rule (included ART status, CD4 count, BMI, and the number of WHO symptoms). The blue line represents the net benefit of W4SS algorithm. The black line represents the assumption that all PLHIV diagnosis with confirmed TB. The grey line represents the assumption that no PLHIV diagnosis with confirmed TB. *PLHIV* people with HIV/AID, *TB* tuberculosis, *W4SS* WHO-recommended four symptom screen

The optimal cutoff for screening TB among PLHIV was the score of 100 (Fig. 5A, sensitivity = 0.93, specificity = 0.35). More information of threshold analysis was in Additional file 4: Table S3. The decision curve analysis for the PTB nomogram among PLHIV was presented in Fig. 5B. The curve showed that the proposed nomogram provided the highest net benefit within the risk threshold range of 10% to 41% compared with the XPHACTOR rule, diagnosis-all, or diagnosis-none scheme. Cost to benefit ratio was from 1:9 to 2:3 in that threshold range. The area under the decision curve was 0.025, higher than the XPHACTOR rule (0.016), W4SS algorithm (0.005), diagnosis-all (0.014), or diagnosis-none (0.00) strategy, respectively.

### Sensitivity analysis

The Additional file 5: Table S4 presented the sensitivity analysis results. The sensitive models with different predictors (by introducing ART status, and substituting CD4 count by ART status) presented no further discriminative information; performed the constant C-index and calibration H–L test results with the favorite nomogram. The AUDC presented no more extra benefit in sensitivity models.

## Discussion

We developed and validated the nomogram to calculate individualized risks, which estimated the active PTB among PLHIV under moderate TB/HIV prevalence. Although its rule was simple and the predictors were accessible, this integrated prediction model accurately discriminated PLHIV who had bacteriological confirmed PTB from those without PTB. The predicted and actual risks were concordances in both primary and validated sets. Furthermore, the nomogram had an additional benefit and outperformed the existing multivariate predict model, suggested the remarkable clinical utility in practice.

The potential impact of this study was the nomogram could be integrated into the disease control policy. The application was the nomogram as a tool to help routine TB control work. The health care provider (HCP) could rapidly calculate risk by hand and improve PTB case findings. In the constrained-resource setting, the bacteriology or molecular test for TB might be unavailable, or the long delay of laboratory test results reported, all of which might lead to the unfavorable consequence of the clinical decision and poor PTB treatment prognosis. The utility of the risk calculator was shortening the diagnosis delay and the time waiting for the TB laboratory tests results. The model predefined the highest probability of active PTB, which was considered the initiation of anti-TB treatment by its high sensitivity.

The significant advantage of the predictive nomogram was laid on its predictors, the PLHIV information contained current CD 4 cell count, the number of WHO screen tool, pulmonary cavity, previous TB history, and smoking status; which enhanced the nomogram accurate and was available and accessible for primary health care unit. The self-reported predictors of symptoms, previous TB history, and smoking status were conveniently collected by HCP. Medical test and examination of CD 4 cell count and Chest X-ray (CXR) was available in most resource-limited situations.

The LASSO method selected the combination of 5 characteristics for the nomogram and met the optimal criterion. Although ART status was an indicator in other clinical models [17, 18], our risk calculator excluded it. In the sensitivity analysis, we added the ART status as an additional predictor. The model with six predictors presented no further discriminative information compared with developed nomogram; The AUDC obtained no more extra benefit for ART status. Another model by substituting CD4 count with ART status performed worse discriminative and AUDC than the developed nomogram. Thus, the HCP could calculate the PTB risk with the above 5 predictors regardless of ART status. For the consideration that in some resource limited countries, CD4 testing was not systematically performed under NTP or the testing might be unavailable, substituting CD4 count by ART status might be an advantage model due to the slight loss of performance in sensitivity model (C-index, 0.70, 95%CI, 0.64–0.76).

The risk calculator integrated with the WHO screening tool could potentially pragmatically impact clinical practice. The latest evidences supported the standpoint that any TB screening tool used alone was insufficient to detect PTB and to excluded no-PTB in PLHIV, because either the low sensitivity or the low specificity of the existing tools [10]. The W4SS algorithm alone has relatively high sensitivity but low specificity (sensitivity = 83%, specificity = 38%) in adults and adolescents living with HIV [15]. A meta-analysis presented the W4SS addition with any abnormal CXR in people on-ART could improve sensitivity to 84.6% but decrease the specificity to 29.8% [37]. Our study supplied a measurement that integrated the WHO tool and other risk predictors, yielded more accurate discrimination and potentially impacts clinical practice. In the ROC and AUC analysis in primary and validation set, the yield of both sensitivity and specificity suggested that the nomogram not only could detect the high PTB risk PLHIV individual but also could be beneficial to rule out the active TB cases.

Another important contribution of this study is that in the cutoff analysis, we defined an optimal threshold score of TB nomogram (cutoff = 100). The interpretation of this would be PLHIV with CD4 count less than 100 cells/mm^3^ was enough to reach 100 points for the nomogram. This would mean that any PLHIV with any symptom and CD4 less than 100 cells/mm^3^ should be systematically investigated for TB. The result was consistent with a recent clinical trial [38]. The high number of PLHIV detected with TB by intensive case finding with urine lipoarabinomannan (LAM), Xpert and chest X-ray regardless on symptom among ART naïve PLHIV with CD4 < 100 cells /mm^3^. Despite the targets proposed by WHO for screening test was 90% for sensitivity and 70% for specificity, the nomogram performed better than other existing tools, which could be applicable for TB screening (sensitivity = 93%, specificity = 35%). There was other use of this nomogram, if the individual with low probability of PTB accompanied by latent TB infection (LTBI) diagnosis, suggested the potential eligibility for the isoniazid preventive therapy.

Previous studies have developed multivariate prediction models for identifying TB among PLHIV. The research by Balcha et al. developed a scoring system of PTB in adult PLHIV in Ethiopia [39]. The model predictors included cough, Karnofsky score, mid-upper arm circumference (MUAC), peripheral lymphadenopathy and hemoglobin. Though the area under curve (AUC) was 0.75, the system was never calibrated and validated. Thus, the generalization ability of the scoring system was unknown. The study by Boyles et al. in South Africa [18] developed an MPM and concluded the lack of response to empiric antibiotics therapy was a strong predictor of TB in PLHIV, C-reactive protein (CRP) adds predictive value only while the second visit and measured after anti-biotics (C-index, 0.75). The sample size was small, and no external validation was done yet. Another research was conducted by Hanifa et al. in South Africa [17]. They developed the XPHACTOR rule, which included ART status, CD4 count, BMI, and the number of WHO symptoms. The rule shown an acceptable C-index (0.75) and good calibration (H–L test *p* = 0.31). We thus did external validation applied the XPHACTOR algorithm to our primary set data. The MPM performance was shown an ordinary discriminative ability (C-index, 0.61) and adequately calibrated by the H–L test (*χ*^*2*^ = 4.37, *p* = 0.49). The deficient validation performance might attribute to the differential study populations and epidemiology risks. More evidences and external validations in different population were needed to verify the generalization and applicability of the XPHACTOR rule. Meta-analysis showed the BMI had a consistent log-linear association with TB incidence in the general population [40]. Higher BMI decreased the HIV-related TB incidence [41]. In our study, both in the primary and validated set, BMI was insignificantly associated with PTB incidence among PLHIV. The reason was that the exposure of high BMI contributed to reducing the PTB epidemic in high and middle-high socio-demographic index (SDI) countries recent years [42]. Meanwhile, the population malnutrition status was still constantly the main risk in the low and middle SDI countries. The demographic and geographical disequilibrium of risk distribution led to the different risk models.

The most important argument for applying the nomogram was based on the interpretation of the individual needs for further treatment or health care. Therefore, to sustain the clinical usefulness of the nomogram, we assessed whether the nomogram decision improved clinical outcomes. The novel method of decision curve analysis was applied to explore the consequence based on threshold probabilities. The indicator of high net benefit derived worthwhile clinical practice. The decision curve showed that if the risk threshold was defined between 10 and 41%, the current nomogram added more benefit than the XPHACTOR rule, W4SS algorithm, diagnosis-all, or diagnosis-none scheme.

The remarkable clinical utility of the nomogram lied in the accurate individualized quantification of the TB risk among PLHIV. By treating the TB risk as a continuum instead of the stratified risk into high-, middle-, or low-risk groups, the study presented TB diagnostic information that could assist the clinical decision-making. Meanwhile, the nomogram provided the clinician and patient with indispensable knowledge to promote the well-informed clinical decision in practice. While the risk threshold in the study was undefined, the nomogram performed better and yield more than existed model and algorithm at any risk threshold. Therefore, the model could be set as the standard for conventional clinic and disease control practice for the rapid screening of TB in the PLHIV, especially in implementing the large-scale active case finding strategy. In addition, PLHIV could revalue and examined the TB risk periodically by using the nomogram themselves. This would benefit the primary prevention for TB among HIV.

Our study has the strength that the well-designed cross-sectional study consecutively included the PLHIV; the representative samples allowed us to develop and validate the practical nomogram. In addition, the study was implemented and reported compliance with TRIPOD guidelines. Furthermore, the novel predictor selection method and decision curve analysis improved the power and interpretation of the research.

However, the study has the limitation that the outcome was based on laboratory bacteriological confirmed PTB, though the updated Ultra outperformed Xpert among PLHIV. The systematic review reported the substantially improved sensitivity for Ultra (90%) compared with Xpert (77%) in PLHIV [14]. This suggested the further study was needed to assess the nomogram by applying the new diagnostic tools. The nomogram did not incorporate some new point of care tests that could potentially have value in screening of TB, include urine LAM and CRP, and these diagnostic tests need further evaluation [15, 43, 44]. Bacteriologically confirmed TB represented only a fraction of all TB cases among PLHIV, and this fraction is even lower when PLHIV are severely immunosuppressed, a prospective cohort using a TB case validation by an independent review committee is needed in further study. Besides, the predictor was self-reported by participants, such as smoking habit and previous TB history, meanwhile, it did not consider of the quantity and duration for the smoking and drinking. The model would be imprecise with inaccurate exposure measurement. Additionally, in many sub-Saharan African countries with more constrained resource, CD4 and CXR test might unavailable, risk factors of TB were dissimilar to Asian, so more evidence was needed for the nomogram generalization in different population. By considering the relative low specificity, future sensitivity analysis should focus on that without CXR or identifying the proportion of patients who reach 100 points without CXR in limited resource countries.

## Conclusions

The study developed and validated a TB nomogram among PLHIV; the simple rule integrated PLHIV’s information: CD 4 cell count, the number of WHO screen tool, pulmonary cavity, previous TB history, and smoking status. The nomogram could benefit in clinical and public health practice and TB intensified case finding strategy, and it should be prioritized for rapid TB screen in PLHIV in a moderate setting.

## Supplementary Information

**Additional file 1. Table S1.** The TRIPOD checklist.

**Additional file 2. Method S1.** The laboratory procedure of TB conformation in PLHIV.

**Additional file 3. Table S2.** The calculation of the variables’ scores.

**Additional file 4. Table S3.** The cutoff and threshold analysis of the tuberculosis nomogram.

**Additional file 5. Table S4.** Sensitivity analysis results of the tuberculosis nomogram.

## Data Availability

All relevant data in this study are freely available in the manuscript as submitted and are included in summary tables and the results section of the manuscript. Dataset is available upon reasonable request to the corresponding author.

## Abbreviations

ART: Antiretroviral therapy
AUC: Area under curve
AUDC: Area under the decision curve
BMI: Body mass index
CRP: C-reactive protein
CXR: Chest X-ray
DCA: Decision curve analysis
HCP: Health care provider
HIV: Human immunodeficiency virus
H–L test: Hosmer–Lemeshow goodness of fit test
IPT: Isoniazid preventive therapy
IQR: Interquartile range
LASSO: The least absolute shrinkage and selection operator
LAM: Lipoarabinomannan
LTBI: Latent TB infection
MPM: Multivariable prediction model
mWRDs: Molecular WHO-recommended rapid diagnostics
MUAC: Mid-upper arm circumference
NTP: National TB program
ROC: Receiver operating characteristic curve
PLHIV: People living with HIV
PTB: Pulmonary tuberculosis
SDI: Socio-demographic index
TB: Tuberculosis
Ultra: Xpert Ultra
WHO: World Health Organization
W4SS: WHO-recommended four symptom screen
Xpert: GeneXpert MTB/RIF assay
