# Supplementary material for: Predicting the risk of active pulmonary tuberculosis in people living with HIV: development and validation of a nomogram
Source: BMC Infect Dis. 2022 Apr 19;22:388. doi: 10.1186/s12879-022-07368-5 (PMC9019965; doi:10.1186/s12879-022-07368-5)
Supplement: Supplementary file 2 — Additional file 2. Method S1. The laboratory procedure of TB conformation in PLHIV. [file 12879_2022_7368_MOESM2_ESM.docx]

**Method S1. The laboratory procedure of TB conformation in PLHIV**

Four sputum samples (two instant spot sputums, the third at night, and the fourth in the following morning) were collected from PLHIV in Yunnan Provincial Hospital of Infectious Disease (YPHID). Two of the sputum samples were examined by sputum smear and semi-nested real-time PCR (Xpert MTB/RIF) in YPHID laboratory. Sputum smear examination adopted the acid-fast staining method (Ziehl–Neelsen staining), each positive result was confirmed by microscopy. Another sample was tested by Xpert MTB/RIF system (Cepheid, USA), the system automatically run to ensure accurate and reliable results.

The remaining two sputum specimens were transferred to the provincial tuberculosis reference laboratory in Yunnan Center for Disease Control and Prevention (YNCDC, 30 kilometres away form YPHID) for further testing. The transfer of strain accordance with the bacterial transfer regulations. YNCDC carried out sputum culture after receiving sputum samples from YPHID. The samples treated with 4% NAOH then inoculated into acidic Roche medium (Löwenstein-Jensen) to observe the results regularly.

All the process above was in accordance with the Chinese National Tuberculosis Programme (CNTP).
