# Supplementary material for: Predicting the risk of active pulmonary tuberculosis in people living with HIV: development and validation of a nomogram
Source: BMC Infect Dis. 2022 Apr 19;22:388. doi: 10.1186/s12879-022-07368-5 (PMC9019965; doi:10.1186/s12879-022-07368-5)
Supplement: Supplementary file 3 — Additional file 3. Table S2. The calculation of the variables’ scores. [file 12879_2022_7368_MOESM3_ESM.docx]

**Table S2 The calculation of the variables’ scores**

| **Factors** |  | **Adjusted**  **Odds Ratio**  ***(exp^(coefficients)^)*** | **95%CI** | ***p*** |  | **Coefficients of regression** | **Rank of coefficients** | **Nomogram score calculation**  ***(score_i_)*** | **Nomogram**  **score *(score_i_)*** | **Variable category** | **Annotation** |
| --- | --- | --- | --- | --- | --- | --- | --- | --- | --- | --- | --- |
| column |  | a | b | c |  | d | e | f | j | k |  |
| CD 4 (< 100 vs. ≥200) |  | 2.55 | (1.44-4.64) | <0.01 |  | 0.935 | 1 | 100 | 100 | dummy variable |  |
| CD 4 (100- 199 vs.≥200) |  | 1.51 | (0.72-3.14) | 0.27 |  | 0.410 | 5 | 100/0.935= *score_i_* /0.410 | 44 | dummy variable |  |
| No of WHO symptoms |  | 1.18 | (1.01-1.42) | 0.05 |  | 0.168 | 6 | 100/0.935= *score_i_* /0.168 | 18 | ordinal variable | For ordinal variable, each 1 unit increase, the score =18*class, e.g. 2 symptoms, the score *i*= 18*2=36 |
| Pulmonary cavity |  | 2.42 | (1.03-5.57) | 0.04 |  | 0.883 | 2 | 100/0.935= *score_i_* /0.883 | 94 | binary variable |  |
| Previous TB history |  | 1.80 | (1.03-3.1) | 0.04 |  | 0.587 | 4 | 100/0.935= *score_i_* /0.587 | 63 | binary variable |  |
| Smoking status |  | 2.23 | (1.43-3.51) | <0.01 |  | 0.804 | 3 | 100/0.935= *score_i_* /0.804 | 86 | binary variable |  |

The calculation of scores:

1^st^ step: get the coefficient of multivariate logistic regression; (column d)

2^nd^ step: ranking the coefficient from the max to min; (column e)

3^rd^ step: set the top of the coefficient score as 100; (column f)

4^th^ step: other variable score were defined as ${score}_{i}=\beta_{i}\frac{100}{\beta_{max}}$ (column j)
