# Supplementary material for: Predicting the risk of active pulmonary tuberculosis in people living with HIV: development and validation of a nomogram
Source: BMC Infect Dis. 2022 Apr 19;22:388. doi: 10.1186/s12879-022-07368-5 (PMC9019965; doi:10.1186/s12879-022-07368-5)
Supplement: Supplementary file 4 — Additional file 4. Table S3. The cutoff and threshold analysis of the tuberculosis nomogram. [file 12879_2022_7368_MOESM4_ESM.docx]

**Table S3 The cutoff and threshold analysis of the tuberculosis nomogram**

| Cutoffs ID | Threshold score of nomogram | Sensitivity | Specificity |
| --- | --- | --- | --- |
| 1 |  | 1.00 | 0.00 |
| 2 | 9 | 0.97 | 0.09 |
| 3 | 27 | 0.97 | 0.14 |
| 4 | 40 | 0.95 | 0.19 |
| 5 | 49 | 0.94 | 0.22 |
| 6 | 58 | 0.94 | 0.24 |
| 7 | 63 | 0.93 | 0.26 |
| 8 | 68 | 0.93 | 0.26 |
| 9 | 76 | 0.93 | 0.27 |
| 10 | 81 | 0.93 | 0.28 |
| 11 | 84 | 0.93 | 0.28 |
| 12 | 92 | 0.93 | 0.32 |
| 13 | 99 | 0.93 | 0.34 |
| 14 | 100 | 0.93 | 0.35 |
| 15 | 102 | 0.90 | 0.37 |
| 16 | 106 | 0.89 | 0.39 |
| 17 | 110 | 0.88 | 0.39 |
| 18 | 114 | 0.87 | 0.39 |
| 19 | 117 | 0.85 | 0.41 |
| 20 | 118 | 0.85 | 0.41 |
| 21 | 120 | 0.83 | 0.47 |
| 22 | 124 | 0.78 | 0.50 |
| 23 | 128 | 0.77 | 0.50 |
| 24 | 133 | 0.76 | 0.51 |
| 25 | 136 | 0.75 | 0.52 |
| 26 | 138 | 0.71 | 0.59 |
| 27 | 142 | 0.71 | 0.61 |
| 28 | 146 | 0.71 | 0.62 |
| 29 | 149 | 0.68 | 0.64 |
| 30 | 152 | 0.68 | 0.64 |
| 31 | 156 | 0.63 | 0.68 |
| 32 | 158 | 0.63 | 0.69 |
| 33 | 160 | 0.61 | 0.69 |
| 34 | 162 | 0.61 | 0.69 |
| 35 | 165 | 0.61 | 0.70 |
| 36 | 167 | 0.61 | 0.72 |
| 37 | 170 | 0.61 | 0.73 |
| 38 | 176 | 0.56 | 0.77 |
| 39 | 180 | 0.55 | 0.78 |
| 40 | 183 | 0.54 | 0.78 |
| 41 | 185 | 0.54 | 0.79 |
| 42 | 186 | 0.53 | 0.80 |
| 43 | 189 | 0.50 | 0.81 |
| 44 | 193 | 0.50 | 0.81 |
| 45 | 197 | 0.50 | 0.81 |
| 46 | 201 | 0.49 | 0.83 |
| 47 | 203 | 0.47 | 0.83 |
| 48 | 208 | 0.43 | 0.84 |
| 49 | 214 | 0.43 | 0.85 |
| 50 | 217 | 0.42 | 0.85 |
| 51 | 219 | 0.41 | 0.85 |
| 52 | 222 | 0.40 | 0.85 |
| 53 | 226 | 0.35 | 0.88 |
| 54 | 230 | 0.34 | 0.88 |
| 55 | 233 | 0.33 | 0.88 |
| 56 | 238 | 0.28 | 0.89 |
| 57 | 244 | 0.22 | 0.93 |
| 58 | 248 | 0.22 | 0.93 |
| 59 | 254 | 0.21 | 0.94 |
| 60 | 260 | 0.13 | 0.96 |
| 61 | 273 | 0.12 | 0.96 |
| 62 | 289 | 0.11 | 0.96 |
| 63 | 296 | 0.10 | 0.96 |
| 64 | 301 | 0.10 | 0.97 |
| 65 | 307 | 0.09 | 0.97 |
| 66 | 313 | 0.08 | 0.97 |
| 67 | 318 | 0.08 | 0.97 |
| 68 | 322 | 0.04 | 0.99 |
| 69 | 329 | 0.03 | 0.99 |
| 70 | 343 | 0.02 | 0.99 |
| 71 | 357 | 0.01 | 1.00 |
| 72 | 379 | 0.00 | 1.00 |
| 73 | Inf | 0.00 | 1.00 |
