# Supplementary material for: Predicting the risk of active pulmonary tuberculosis in people living with HIV: development and validation of a nomogram
Source: BMC Infect Dis. 2022 Apr 19;22:388. doi: 10.1186/s12879-022-07368-5 (PMC9019965; doi:10.1186/s12879-022-07368-5)
Supplement: Supplementary file 5 — Additional file 5. Table S4. Sensitivity analysis results of the tuberculosis nomogram. [file 12879_2022_7368_MOESM5_ESM.docx]

**Table S4 Sensitivity analysis results of the tuberculosis nomogram**

| Sensitivity analysis | Factors | Adjusted  Odds Ratio | 95%CI | | *p* |  | C-index | 95% CI |  | H-L test | |  | AUDC |
| --- | --- | --- | --- | --- | --- | --- | --- | --- | --- | --- | --- | --- | --- |
|  |  |  | low | high |  |  |  |  |  | *χ^2^* | p |  |  |
|  | Intercept | 0.03 | 0.01 | 0.10 |  |  |  |  |  |  |  |  |  |
| Sensitivity Model 1  Added ART status to the nomogram | CD 4 (< 100 vs. ≥200) | 2.28 | 1.26 | 4.22 | <0.01 |  | 0.72 | 0.66-0.77 |  | 3.57 | 0.61 |  | 0.0267 |
|  | CD 4 (100- 199 vs.≥200) | 1.39 | 0.65 | 2.93 | 0.38 |  |  |  |  |  |  |  |  |
|  | No. of WHO symptoms | 1.17 | 0.98 | 1.40 | 0.09 |  |  |  |  |  |  |  |  |
|  | Pulmonary cavity (yes *vs.* no) | 2.53 | 1.07 | 5.89 | <0.05 |  |  |  |  |  |  |  |  |
|  | Previous TB history (yes *vs.* no) | 1.92 | 1.09 | 3.35 | <0.05 |  |  |  |  |  |  |  |  |
|  | Smoking status (current *vs.* never) | 2.18 | 1.39 | 3.43 | <0.01 |  |  |  |  |  |  |  |  |
|  | ART (yes *vs.* no) | 0.70 | 0.43 | 1.12 | 0.14 |  |  |  |  |  |  |  |  |
|  |  |  |  |  |  |  |  |  |  |  |  |  |  |
| Sensitivity Model 2  Substituting CD 4 count by ART status to the nomogram | Intercept | 0.06 | 0.02 | 0.18 |  |  |  |  |  |  |  |  |  |
|  | No. of WHO symptoms | 1.25 | 1.05 | 1.49 | <0.05 |  | 0.70 | 0.64-0.76 |  | 3.63 | 0.60 |  | 0.023 |
|  | Pulmonary cavity (yes *vs.* no) | 2.84 | 1.21 | 6.57 | <0.05 |  |  |  |  |  |  |  |  |
|  | Previous TB history (yes *vs.* no) | 1.94 | 1.10 | 3.36 | <0.05 |  |  |  |  |  |  |  |  |
|  | Smoking status (current *vs.* never) | 2.06 | 1.32 | 3.22 | <0.01 |  |  |  |  |  |  |  |  |
|  | ART (yes *vs.* no) | 0.59 | 0.37 | 0.93 | <0.05 |  |  |  |  |  |  |  |  |

Abbreviations: AUDC,area under the decision curve; ART, antiretroviral therapy; H-L test, Hosmer-Lemeshow goodness of fit test
